# Acknowledgement to Reviewers of the International Journal of Environmental Research and Public Health in 2013

**DOI:** 10.3390/ijerph110302521

**Published:** 2014-02-28

**Authors:** IJERPH Editorial Office

**Affiliations:** MDPI AG, Klybeckstrasse 64, CH-4057 Basel, Switzerland

The editors of the *International Journal of Environmental Research and Public Health* would like to express their sincere gratitude to the following reviewers for assessing manuscripts in 2013:
Aasvang, GunnHaber, SteveOsborne, KatyAbballe, AnnalisaHadjichristodoulou, ChristosOscar, ThomasAbreu, MauroHagen-Zanker, AlexOsek, JacekAbughosh, Susan M.Hager, Erin R.Otsuka, YasumasaAbuknesha, Ramadan A.Hahn, JoanOtsuki, MichikoAcharya, LalatenduHahn, YoujinOttosson, Jakob RydAdams, James B.Hailey, DavidOwusu, AndrewAdams, Monica L.Hale, LeighPaciorek, ChrisAdeseun, GbemisolaHall-Mendelin, SonjaPadmanabhan, VasanthaAdlam, Timothy D.Hall, Pamela R.Paek, JiheumAdler, Meghan E.Halonen, Jaana I.Pagán, José A.Afonso, José A.Halperin, William E.Page, KristenAgalakova, NataliaHalsall, JamiePalacios-Ceña, DomingoAittasalo, MinnaHambach, RamonaPalmiere, CristianAkhalkatsi, MaiaHamer, Gabriel L.Palù, GiorgioAlbert, Steven M.Hamer, MarkPampaka, DespinaAlencar, Carlos HenriqueHammitt, JamesPan, Jen-YiAllanson, PaulHampson, David J.Panico, LidiaAllen, HarrisHancock, TrevorPapa, AnnaAllerberger, F.Hanewinkel, ReinerPapadopoulos, AndrewAltaweel, MarkHanke, WojtekPappas, SteveAlverson, Dale C.Hanna, LizPark, AndrewAlves, ElisabeteHansen, Claus D.Park, KyongAmarasena, NajithHanson, TimothyPartonen, TimoAmaro, InesHarada, MasafumiPartridge, BradleyAmin, RaidHarding, JohnPatil, Grete GrindalAnafi, PatriciaHariharan, HarryPatnaik, JenniferAncona, NicolaHarjutsalo, ValmaPatwary, Masum A.Andersen, Helle R.Harlan, SharonPaul, MathildeAndersen, Lars L.Harley, DavidPaunović, KatarinaAnderson, PeterHarony, HalaPawlas, NataliaAnderson, Peter D.Harper, SherileePawloski, Lisa R.Anenberg, Susan C.Harrington, JohnPawłowska-Góral, KatarzynaAñez, GermánHarris, Anne M.Payment, PierreAngelopouloua, ElliHarris, MatthewPayne, SarahAnopa, YuliaHartig, TerryPearce, Dora ClaireAntunes, PatríciaHarvey, RogerPearce, Elizabeth N.Appel, WyatHashsham, Syed A.Pearson, Jennifer L.Ara, IgnacioHaslam, Divna M.Pearson, LeeArboleda, SairHasselberg, MariePedersen, Charlotte GjørupArcury, Thomas A.Hauer, KlausPedley, StephenArgos, MariaHaukka, KaisaPedrotti, JairoArimilli, SubhashiniHawley-Hague, HelenPeel, Jennifer L.Ariño, CristinaHayes Jr., DonPeen, JaapArmstrong, Philip M.He, JiePekmezovic, TatjanaArroyo-Cobo, Jose ManuelHe, YonghuaPenney, Tarra L.Asakawa, AkihiroHe, YongqingPennig, SibylleAslam, MueenHealy, Genevieve N.Perez-Ríos, MónicaAslan, AsliHeaviside, ClarePerles-Roselló, María JesúsAtsuo, KishimotoHeilig, Charles M.Perry, Sara JansenAttanasio, LauraHein, AndreasPetersen, ArndAttfield, MichaelHendrickx, GuyPetersen, ElijahAuchincloss, Amy H.Hendryx, MichaelPetersen, EskildAumann, CraigHenkin, Alan B.Petersen, SibylleAung, MayHennrikus, DeborahPetrass, LaurenAunger, RobertHenriksen, LisaPetrosillo, IreneAurrekoetxea, Juan J.Hens, NielPetrović, VladimirAustin, LaurelHeo, JinseokPfeiler, TamaraAutenrieth, ChristineHeo, JonghoPheasant, RobertAvogo, Winfred AweyireHernandez-Jover, MartaPhillips, JulieAyo-Yusuf, Olalekan A.Hernando Sebastián, VictoriaPicano, EugênioAzmitia, Efrain C.Heukelbach, JorgPichini, SimonaBabalola, Stella O.Hines, Cynthia J.Pierce, Lisa M.Backhans, AnnetteHirsch, Alec J.Pilgrim, DavidBagella, SimonettaHiscock, RosemaryPillay, VinessBagramian, RobertHitzhusen, FrederickPinchevsky, GillianBaker, Mary KateHo, Sai YinPira, EnricoBakonyi, TamasHobst, LeonardPires, José Carlos MagalhãesBal, WojciechHochrainer-Stigler, StefanPitkänen, TarjaBalcazar, HectorHoff, Dale J.Platonov, Alexander E.Balk, Sophie J.Hofmann, BjørnPohanka, MiroslavBallbè, MontseHofmeister, Erik K.Polimeni, AntonellaBammann, KarinHogan, AnthonyPolosa, RiccardoBanach, MaciejHolbrook, Michael R.Pols, JeannetteBannon, DesmondHolick, Michael F.Pompili, MaurizioBao, YongpingHollen, VeraPonce, CarmenBarber, Sarah L.Holzel, ChristinaPonnet, KoenBarbieri, FlaviaHondula, DavidPontremoli, RobertoBarclay, KieronHong, Jun SungPorteous, NualaBarlow, Karen MariaHooker, BrianPośpiech, BeataBarnes, Philip L.Hoppin, JanePotashkin, JudithBarnett, Tracey E.Hoppin, PollyPottage, ThomasBarreca, AlanHornikx, MaartenPoulsen, Henrik E.Barrera, RobertoHorton, GraemePowers, Ann M.Bartold, MarkHosgood III, H. DeanPrado, Gustavo FaibischewBarzon, LuisaHoshiko, SumiPrettner, KlausBashshur, Rashid L.Hoskins, John A.Preve, NikolaosBasu, RupaHoughton, AdelePribis, PeterBatchelor, FrancesHoward, Matthew O.Price, BronwynBaumgarth, NicoleHowe, PeterPrincipato, MaryannBaverstock, KeithHoyert, Donna L.Prins, Pier J. M.Bayat, FariborzHrubá, DrahoslavaProper, KarinBayer, RonaldHsiao, Hui-WenPrus, StevenBeaver, Kevin M.Hu, BrianPulkki-Råback, LauraBecoña, ElisardoHu, XuefeiPurkis, SteveBedore, MelanieHuang, GordonPurshouse, RobinBeebe-Dimmer, JenniferHuang, JinPutnam, MichelleBeenackers, Marielle A.Huang, Jiun-HauQi, FengBefort, ChristieHuang, LihuaQin, Hua-PengBehson, Scott J.Huang, Wei-ShinQuantin, XavierBeilhe, Leila BagnyHudson-Edwards, KarenQuinn, Brian C.Belasco, EricHuen, KarenRahman, MahfuzarBeldowski, JacekHughes, KarenRalph, Stephen J.Bellatreche, LadjelHuijts, TimRamos-Jorge, MariaBellomini, RaffaellaHunt, AlistairRatschen, ElenaBellou, AndrianaHunter, Bronwyn A.Rayner, GeofBenbenishty, RamiHunter, RuthReady, Paul D.Benfield, JacobHuntington, HenryReagan, Krystle L.Benjamin-Garner, RubyHurme, MarkkuRebolj, MatejkaBennett, JamieHutchinson, AmandaReddy, MadhaviBere, EllingHwang, Cheng-AnReefhuis, JennitaBerg, Christine D.Hyder, Adnan A.Regidor, EnriqueBerntsen, SveinungHystad, PerryReid, AlisonBerry, E. HelenIamarino, MarioReiner, Robert C.Berry, PeterIanieri, AdrianaReinhold, SteffenBertherat, EricIchiba, MasayoshiReisen, William K.Berwick, MarianneIkem, AbuaRen, Wei (Vivian)Bett, BernardJacinto, CelesteReniers, GenserikBhandari, MediniJacobs, DavidRethorst, Chad D.Bhunia, ArunJahnke, Sara A.Reutfors, JohanBianchi, FabrizioJakovljevic, MihajloRey, GrégoireBierman, KarenJakubovics, Nicholas S.Rhee, Hae-ChunBingham, JohnJakus, PaulRheinberger, Christoph M.Bisson, Mary A.Jalava, KatriRhona, JulienBiziuk, MarekJambrosic, KristianRiccardo, FlaviaBjörkenstam, EmmaJanczukowicz, WojciechRiccio, AngeloBlanco, CristinaJang, Hyung-KwanRice, ScottBlanford, JustineJanson, StaffanRice, TomBlank, MichaelJardine, AndrewRichardson, RichardBlitvich, BradJawad, MohammedRickard, Laura N.Bloch, MicheleJay, OllieRigdon, SteveBlunt, RuthJennett, PennyRijnaarts, HuubBobylev, NikolaiJeon, ByeonghwaRiley, EdwardBocchiccio, FrancescoJeon, Jin YongRiordan, JamesBockerman, Petri RikharJeong, Hyo-JoonRíos-Bedoya, Carlos F.Bonevski, BillieJeong, Seok HoonRoberts, HelenBoniface, DavidJepsen, Martin RudbeckRoberts, SarahBoot, CécileJesdale, BillRobinson, GarethBopp, MelissaJetter, James J.Robson, MarkBorchard, Mark A.Jevšnik, MojcaRocheleau, Gregory C.Borja-Aburto, Víctor HugoJex, Aaron R.Rochlin, IliaBoroushaki, Mohammad TaherJiang, HongchenRodda, NicolaBorse, Nagesh N.Jiang, YuRodrigues, IreneBostelman, RogerJimenez-Cisneros, BlancaRodrigues, Jean-MarieBoudrez, HedwigJiménez, Víctor SeguraRodríguez-Martín, Jose AntonioBoyce, WalterJing, ChuanyongRodriguez, Rudolph A.Boyd, Robert S.Jo, Wan-KuenRoe, JennyBradstreet, James JeffreyJohansen, CherylRolle, Italia V.Brand, SergeJohansson, AnnaKarinRollinson, DavidBrandsema, PetraJohansson, PerRoman, Henry A.Braubach, MatthiasJohn, Dolly A.Romero, Miguel SánchezBrault, Aaron C.Johnson, AaronRonda-Perez, ElenaBreitner, SusanneJohnson, Brian J.Rondinelli, ConcettaBrenner, IngridJohnson, GregRosário, RafaelaBriggs, DavidJohnson, Linda S. B.Rose, JenniferBrink, MarkJohnston, Carl GordonRosen, BarryBroadbent, Jonathan M.Johnston, Douglas M.Rosenkranz, Richard R.Brodie, Ian M.Johnston, Jill E.Rosenquist, HanneBronsvoort, MarkJones, AnneRossignol, DanielBrooks, AntoneJones, RayRoy, AmandaBrough, HelenJönsson, KristinaRu, ShaoguoBrown, HelenJordan-Marsh, MaryaliceRuano-Ravina, AlbertoBrowne, MarilynJorstad-Stein, Ellen C.Rubio, Doris McGartlandBrunette, MaryJoubert, KarinRudatsikira, EmmanuelBrunetti, VirgilioJu, Hwang WonRuecker, Norma J.Brussoni, MarianaJulian, ErnestRuel, ErinBuckwalter, J. GalenJun, Young-ShinRuiz-Bustos, EduardoBudke, Christine M.Jung, Myung-ChaeRuiz, JoaquimBugiardini, RaffaeleJurewicz, JoannaRull, RudyBuja, AlessandraKaang, Bong-KiunRussell-Mayhew, ShellyBujnowska-Fedak, Maria MagdalenaKahan, DavidRuzauskas, ModestasBurd, LarryKaiiry, DahliaRyan, Patrick H.Burd, MartinKalengayi, Faustine NkuluSabbath, ErikaBurdorf, AlexKaltenbach, MartinSabri, BushraBurk, ToniaKamp, David W.Saddleson, MeganBurrough, EricKampel, MartinSadler, RichardBurrows, StephanieKang, Choong-MinSáfadi, Marco A. P.Bus, Boudewijn A. A.Kang, SanghoonSainz, CarlosBush, TerryKang, YanmingSáiz, Juan-CarlosBusova, MilenaKarakitsios, SpyrosSakakibara, MasayukiButler, Danielle C.Karanikolos, MarinaSalmain, MicheleByass, PeterKaranis, PanagiotisSalminen, SimoByrd-Bredbenner, CarolKarlsson, MartinSambrie, VittorioByrne, DominicKäsbohrer, AnnemarieSánchez, DavidCabral, João P. S.Kasper, ChristineSancho-Garnier, HeleneCai, BoKatanoda, KotaSanmiquel, LluísCalderaro, AdrianaKatayama, HiroyukiSano, DaisukeCalderón-Garcidueñas, LilianKatbamna, BhartiSantos, Maria PaulaCalistri, PaoloKatner, AdrienneSantos, Ubiratan De PaulaCalvert, Amanda E.Kaufman, James S.Sarwer, David B.Cama, JordiKawamura, TakashiSass, JenniferCampbel, LauraKayhanian, MasoudSato, AzusaCampbell, DanielKeener, TimSato, HirokiCampbell, Kathleen C. M.Keeshin, BrooksSato, YukitaCampos, Guillermo LopezKefauvera, Shawn C.Sauleau, Erik-AndréCan, ArnaudKeizer, InekeSauni, RiittaCanfield, Mark A. CanfieldKelepertzis, EfstratiosSavini, GiovanniCantón-Cortés, DavidKemptner, DanielSchenk, NielsCao, KaiKerminen, Veli-MattiSchillo, BarbaraCapra, ValeriaKern, Janet K.Schindler, ChristianCaputo, TullioKerret, DoritSchlomit, PazCapuzzo, MauriziaKershaw, Kiarri N.Schmidt, AnjaCardel, MichelleKesavaraju, BanugopanSchmitz, NorbertCaroli, SergioKessler, Ronald C.Schoen, CathyCarpenter, DavidKey-Schwartz, RosaSchoueri-Mychasiw, NourCarpentier, BrigitteKhan, ArifullaSchrauzer, GerhardCarrera, María VictoriaKiem, AnthonySchuck, AmieCarrero, Jose AntonioKile, MollySchug, JoannaCarrieri, MarcoKim, HeonSchultz, AnnetteCarrieri, VincenzoKim, HoSchultz, StephenCarson, KristinKim, Hun KyungSchulz, Jennifer J.Carter, Megan A.Kim, HyunSchuurman, NadineCarvajal, ScottKim, JeungimSchwaiger, KarinCassidy-Bushrow, Andrea E.Kim, Ki-HyunSchwebel, DavidCasson, KarenKim, Sun S.Schweizer, Vanessa J.Castellano, ImmacolataKimura-Ono, AyaScialli, Anthony R.Chagnes, AlexandreKing, EoinScotch, MatthewChaiton, MichaelKipen, Howard M.Scott-Sheldon, Lori A. J.Chan, Ding-ChengKira, MariScott, ElizabethChandwani, SulachniKirkpatrick, SharonScotton, Carol R.Chang, Ching-ChengKishida, NaohiroSebastiani, PaolaChang, Hung-HaoKjellsson, GustavSeenan, ChrisChari, RamyaKlaschka, UrsulaSeguin, RebeccaChariot, PatrickKlein, ElizabethSella, LucaCharles, KatrinaKleinsteuber, SabineSelman, CarolCharlton, Brittany M.Klimas, JanSelya, ArielleChatignoux, ÉdouardKnobf, M. TishSembajwe, GraceChatzipanagiotou, StylianosKnopper, LorenSengor, SevincChau, Chi-KwanKo, Jiunn-LiangSenthilselvan, AmbikaipakanChaves, Luis FernandoKoehly, Laura M.Serrano, AntoniChen, Chi-FengKoelemay, Mark J. W.Serrano, EmilioChen, KepingKogut, Michael H.Setton, Eleanor M.Chen, Kow-TongKoivisto, JoonasSexton, KenChen, LeiKolehmainen, MikkoShadoin, AmyChen, Pei-ShihKoletsi-Kounari, HaroulaShakoori, Abdul RaufChen, Szu-ChiehKolovos, AlexanderShanahan, FergusChen, Wan-QingKomar, NicholasShapiro, KarenChen, WeiHongKominkova, DanaSheffield, Perry E.Chen, XinGuangKong, In ChulShen, ZhangquanChen, ZhuoKonishi, EijiShenawy, Nahla S. ElCheong, Hae-KwanKoo, MalcolmShepherd, DanielCherrington, Nathan J.Koohsari, JavadSherman, RecindaCheung, Chau-KiuKorfmacher, KatrinaShi, XianglinChiabai, AlineKormas, KonstantinosShi, XunChiara, PanariKorsak, DorotaShiao, Yih-HorngChilenski, Sarah M.Korzeniewska, EwaShibayama, KeigoChin, Dal LaeKosterink, StephanieShiels, Aaron B.Chiou, Hung-YiKostkova, PattyShiyko, MariyaChipps, JenniferKostova, DelianaShoff, CarlaChirizzi, DanielaKotzalidis, Giorgio D.Short, Camille E.Cho, Kyoung-ImKoufi, VassilikiShortt, Niamh K.Chobert, Jean-MarcKousoulas, Konstantin G.Shriar, Avrum J.Choi, KelvinKovalenko, KasparsSiegert, RichardChomistek, Andrea K.Kovalskyy, ValeriySiever, Larry J.Chomutare, TaridzoKozyrskyj, Anita L.Silbergeld, EllenChou, Yiing-JengKrakat, NiclasSilley, PeterChow, Maria Yui KwanKramer, Laura D.Silva, LígiaChow, Winston T. L.Kranz, Ashley M.Simpson, JeanChristensen, HenrikKreichauf, SusanneSims, JaneChristophe, DanielKrienert, Jessie L.Sims, LesChristophe, WaterlotKristiansen, JesperSinclair, RyanChuang, Chun-YuKristjuhan, ÜloSintonen, SannaChuang, Kai-JenKroesen, MaartenSkoczyńska, AnnaChuang, Ting-WuKrogsbøll, Lasse T.Skön, Jukka-PekkaChung, Chung-YiKrupinski, ElizabethSkouloudis, Andreas N.Chung, StephanKrysinska, KarolinaŠkrbić, BiljanaCiccozzi, MassimoKudirkienė, EglėSkudutyte-Rysstad, RasaCikajlo, ImreKühner, DietrichSlaymaker, TomClaes, NereeKukizaki, MasatoSloan, RobertClaeson, Anna-SaraKulesza Jr., Randy J.Slotterback, Carissa SchivelyClarke, MalcolmKull, IngerSmani, YounesClarkson, Peter M.Kundi, MichaelSmith, BradleyClayton, SusanKungskulniti, NipapunSmith, ClaireCliff, DavidKuo, Shu-ChenSmith, MarkClougherty, Jane E.Kvaavik, ElisabethSmyrnowa, JulijaCocco, PierluigiKwapisz, AgnieszkaSohl, TerryCochran, Joshua C.Lacerda, Silvia H.Solcan, CarmenCoelho, Ana CláudiaLachmann, NicoSoljak, MichaelCohen, ArtLadeau, Shannon L.Solo-Gabriele, HelenaCohen, Mitchell D.Lagrosen, YvonneSon, Nguyen-ThanhCohen, Samuel M.Lai, Chih-HoSong, RhayunColdea, GheorgheLai, DejianSong, YongColeman, BrendaLai, Ka-ManSosa, ManuelCollings, PaulLamb, JosephSospedra, JavierCollins, Marietta H.Lamm, Steven H.Sousa, Sofia I. V.Collins, Timothy W.Lämmle, LenaSovio, UllaColwell, DougLampman, RichSoyer, PeterCongdon, PeterLando, AmySpangler, JohnConger, ScottLando, HarrySpanò, CarmelinaCook, Joseph H.Landschoot, SofieSparrow, RobertCooper, DavidLang, KatrinSpector, LoganCooper, RalphLangley, KateSpengler, John O.Cordioli, PaoloLapeyrouse, LisaSrivastava, ShouryadeepCormac, IreneLassnig, HeimoStaddon, PhilipCormier, YvonLau, MichaelStadler, HermannCornelsen, LauraLaurent, ÉloiStagnitti, KarenCoronato, AntonioLaurier, DominiqueStamatis, NikolaosCoss, RichardLavelle, BridgetStanford, KimCosta, MaxLavoué, JérômeStansbury, KimCostea, Daniela ElenaLawn, SharonStansfeld, StephenCoulton, Claudia J.Lawson, Andrew B.Stawiarska-Pięta, BarbaraCox, ChadLayte, RichardStefanelli, DarioCox, GeorginaLazos, Evangelos S.Stefanogiannis, NikiCoyne, MarkLazuras, LambrosStein, GregorCrane, PatriciaLe Strat, YannSteiner, FrederickCroezen, SimoneLeal, Luz O.Steinmann, PeterCronkite, Ruth C.Leão, Pedro N.Stelzer, JiriCruz-Mendoza, IreneLee, Duk-HeeStemplewski, RafalCruz, Adriano G.Lee, Hu JangStepanov, IrinaCulberson, KurtLee, Jeong-ChaeStichler, Jaynelle F.Cullinan, PaulLee, Joseph G. L.Stockton, JemimaCummings, David E.Lee, KylieStokkeland, KnutCurrid, ThomasLee, Lisa M.Stone, DebD'anna-Hernandez, KimberlyLee, Sue Ann S.Strandh, MattiasD'Arcy, ShonaLee, Won JinStrobel, NatalieDa Cunha, Moore K. DiasLee, Won-JeongStrohmeier, DagmarDai, HepingLehucher-Michel, Marie-PascaleStyers, Diane M.Dakulincová, ZuzanaLei, YunpingSu, ChangDale, AnnLeicester, AndrewSuenaga, HikaruDalton, Alice M.Leisner, Jorgen J.Suggs, SuzanneDalton, Harry R.Lemke, KlausSultan, ZuraimiDamashek, AmyLenz, BerndSumner, Walton IiDamiani, GianfrancoLeon, NatalieSun, HongminDanielsen, Tor ErikLeong, DavidSun, QinghuaDarbra, Rosa-MariLepak, JesseSun, Ya-YenDavies, RobertLeslie, Timothy F.Suñol, CristinaDavis, MarkLessman, CharlesSuominen, TarjaDavis, Meghan F.Levine, HagaiSvensen, ErikDe Carvalho Ponce, JulioLevy, JonathanSwanberg, Jennifer E.De Coensel, BertLevy, KarenSweeney, Daniel W.De França, Elvis JoacirLewis, FerdinandSykes, CatherineDe Grey, AubreyLewis, Fraser I.Szabó, SzilárdDe Haan, CarolineLi, FangSzymańska, JolantaDe Hert, MarcLi, Fang-BaiTakahashi, MasayoshiDe Hert, PaulLi, HuabinTakeshita, HaruoDe La Torre Reoyo, AnaLi, KaigangTalbert-Slagle, KristinaDe Leo, GianlucaLi, LongjianTalbot, PrueDe León, Byron CalguaLi, SenTandlich, RomanDe Luca, PatrickLi, YingxiaTang, ZhenghongDe Munter, JeroenLi, YonghuaTasi, Su-YingDe Oliveira Fernandes, EduardoLi, ZhigangTaylor, SallyDe Sario, ManuelaLikotrafiti, EleniTehranifar, ParisaDe Souza Vieira, Luiza Jane EyreLim, Min K.Telfair, JosephDebess, Emilio E.Lin, ChadTer Kuile, BennoDecker, Sandra L.Lin, HuiTham, Kwok WaiDel Razo, LuzLin, Kwan-HwaThastum, MikaelDemeke, TigstLin, LinTheodoropoulos, GeorgiosDemiroglu, CenkLin, Yuan-ChungThomas, DavidDesenclos, Jean-ClaudeLincoln, Jennifer M.Thomas, David PDeserno, Thomas M.Linder, StephenThompson, Richard E.Devís-Devís, JoséLindsay, DavidThompson, WendyDevleesschauwer, BrechtLindström, MartinThomson, MurrayDevlieger, RolandLiu, Chi-HungTian, GuoxunDewan, AshrafLiu, Ching-KuanTian, LinweiDewe, PhilipLiu, Hai-YingTitze, SylviaDickerson, FaithLiu, HongbinTobita, KimimasaDifranza, JosephLiu, ShuTodd, EwenDiGiacomo, MichelleLiu, YandiToft, GunnarDiraco, GiovanniLiu, YangTokajian, Sima T.Dirks, Kim N.Liu, YaolinTol, Richard S. J.Dismuke, LibbyLivingston, Kimberly A.Tommasi, FrancaDittmer, DirkLoaiza-Bonilla, ArturoTopitzes, DimitriDittrich, RalfŁobacz, AdrianaTorija, Antonio J.Diuk-Wasser, Maria A.Lobb, RebeccaToscano, WilliamDivaris, KimonLøbner-Olesen, AndersTouboul Lundgren, PiaDjenane, DjamelLogue, Catherine M.Tousignant, MichelDjietror, GodwinLonardo, AmedeoTrail, FrancesDjomo, Sylvestre NjakouLongo, SoniaTralau, CatherineDo, D. PhuongLönnerfors, CelineTran, Anh N.Dobke, Marek K.López-Navarro, Miguel ÁngelTrasande, LeonardoDockery, MichaelLoprinzi, Paul D.Triassi, MariaDoctor, HenryLoss, Scott R.Truax, BarryDodson, RobinLoughnan, MargaretTrudgett, AlanDomínguez, AngelaLourenço, Roberto WagnerTsai, Hui-JuDong, Cheng-DiLowenstein, Lisa M.Tsai, JackDong, Xin QiLu, HongTsai, Perng-JyDons, EviLu, JiafangTsai, TedDorevtich, SamLu, WeizhenTsanas, AthanasiosDouglas, IanLu, XinweiTseng, Chin-HsiaoDouterelo, IsabelLucchini, SachaTsimbos, CleonDu, HongfeiLucian Szasz, PaulTsuchiya, Kenji J.Du, LiboLucio, Paulo SergioTu, JunDubrova, Yuri E.Łuczkiewicz, AnetaTurell, Michael J.Ducret-Stich, Regina E.Luginaah, IsaacTuvesson, HannaDuffy, Sonia A.Lukas, MichaelTyrväinen, LiisaDumètre, AurélienLuo, Zhong-ChengTzelepis, FloraDunbar, James D.Luque, JohnUlbert, SebastianDunbar, Michael StephenLusk, AnneUnger, JenniferDunière, LysianeLynne, DawkinsVäisänen, LiisaDunn, GemmaLyons, MinnaVan Beijnum, Bert-JanDunst, Carl J.MacCalman, LauraVan Broekhuizen, PieterDupuis, AlanMackinnon, GillianVan Cauwenberg, JelleDurey, AngelaMaggi, StefaniaVan Delden, HedwigDurrani, HammadMaglaveras, NicosVan den Borst, BramDykman, Lev A.Magnone, EdoardoVan Den Branden, SigridEasterling, DougMagori, KrisztianVan Den Hurk, Andrew F.Ebi, KristieMain, PenelopeVan Der Bij, Akke K.Eckhart, Vincent M.Malec, BrianVan Der Kooij, DickEdwards, BenMalkhazova, SvetlanaVan Der Poel, WimEgli, ThomasMalliarou, MariaVan Ewijk, ReynEichner, MartinMalmusia, DavideVan Hoof, Joris J.Eisenberg, DanielManaia, Célia M.Van Kamp, IreneEisenberg, JosephMancianti, FrancescaVan Kippersluis, HansEkman, BjörnMancini, LauraVan Kleef, RichardElenge, MyriamManduca, PaolaVan Laer, LutElizur, ArnonMangalore, RoshniVan Orden, KimberlyElliott, Michael B.Manios, StavrosVan Renterghem, TimothyElstad, Jon IvarMannino, David M.Van Rie, AnneliesEnglish, PaulMansoorc, Mohammad AzamVan Ruijven, Bas J.Erazo, MarciaMarcucci, FabrizioVan Sluijs, EstherErhart, LauraMarengoni, AlessandraVan Zandwijk, NicoErreygers, GuidoMargolis, HeleneVandebriel, Rob J.Escobedo, Francisco J.Marin, DanielaVandelanotte, CorneelEscoffery, CamMaritz, Gert S.Vanderplasschen, WouterEvans, KenMarkevych, IanaVandoros, SotirisEves, FrankMarkides, Kyriakos S.Vargo, JasonEysel-Gosepath, KatrinMarquis-Faver, CatherineVaughan, Jefferson A.Fabian, PatriciaMarsiglia, FlavioVeiga, LeneFagan, AbbyMartin, BarbaraVerloigne, MaïtéFallin, AmandaMartinez, CristinaViana, MarFang, Chen-LingMartinez, Nelda C.Vieux, Baxter E.Fang, HuiMarzano, MariaViladrich, AnahíFann, NealMasala, GiovannaVilhelmsson, OddurFarr-Wharton, RodMasfety, Viviane KovessVillanueva, VicentFaruque, FazlayMassányi, PeterVitali, MatteoFederico, BrunoMasuda, NaokiVlachokostas, ChristosFelföldi, TamasMathiesen, Amber M.Vollmer, SebastianFeng, PeterMatsuda, ShingoVon Essen, SusannaFensli, RuneMattes, AlVon Mühlendahl, Karl ErnstFergusson, David M.Matthews, CharlesVon Wehrden, HenrikFernandez-Ballesteros, RocíoMatthies, FranziskaVoulvoulis, NickFerrario, Marco M.Matzarakis, AndreasWade, TimFerro, RobertoMaurer, JohnWadsworth, Daniel P.Fesus, GabriellaMawson, AnthonyWahlbeck, KristianFickenscher, HelmutMay, FionaWalerian, ElżbietaFigueira, EtelvinaMayne, SusanWalker, AshleyFinch, MeghanMayner, LidiaWalker, JimmyFinkelstein, FedricMazumdar, SoumyaWallander, JanFinlayson, JanetMcaughey, JohnWallmann-Sperlich, BirgitFinley, RichardMcBride, Graham B.Wampler, PeterFischer, ArnoutMcclure, PeterWan, Man-PunFischer, DominikMccowan, LesleyWang, FahuiFischer, GloriaMcCoy, Sarah J. BreeseWang, Heng-ShanFischer, Joachim E.McCrossan, Brian A.Wang, JikunFisher, Gwenith G.McDiarmid, Melissa AWang, ShengruiFisk, Malcom J.McDonald, TedWang, YuanpengFlemming, Hans-CurtMcDonough, SuzanneWang, Zhong-QuanFleury, AnthonyMcGuckin, MichaelWarburton, Darren E. R.Florence, Curtis S.McKallip, Robert J.Ward, Michael P.Fonnum, FrodeMcKay, RuthWargocki, PawelFonseca, Dina M.McLafferty, SaraWarnakulasuriya, SamanFont, LaiaMcLellan, Deborah L.Warren, JimFord, TimothyMcMillen, RobertWarren, JoshuaFornalski, Krzysztof WojciechMcNally, RichardWarren, PaigeFornaro, AdalgizaMcPhedran, SamaraWarriner, KeithForsberg, BertilMcSpirit, StephanieWasem, JuergenForsythe, SeteveMedek, DanielleWaters, Andrew J.Foster, JohnMehta, Tara G.Waterworth, Eva LindhFostervold, Knut-IngeMeinke, DeannaWatt, Toni TerlingFoubert, ImogenMelander-Wikman, AnitaWatts, GregFozard, James L.Melke, JonasWebb, Cameron E.Frank, AndrewMendenhall, IanWebb, ChristianFranklin, RichardMéndez, Claudio A.Weber, GermainFransson, EleonorMenezes-Filho, Jose AntonioWeber, MiriamFranzblau, AlfredMenozzi, DavideWei, BinnianFranzetti, LauraMenvielle, GwennWeinreb, AlexanderFrederick, TylerMetcalf, PatriciaWeinstein, PhilipFreeman, MattMeyer, ElisabethWeitzman, Michael L.Freeman, TobyMichaelakis, AntoniosWen, Kuang-YiFreestone, MarkMichelin, Marcia A.Wen, Li-MingFreiberger, EllenMichimi, AkihikoWeng, Chih-YuanFrenken, MelinaMiles, Lisa M.Werler, MarthaFriesen, Kristina M.Miller, GloriaWertheimer, AlbertFroehlich-Grobe, KatherineMin, Kyung RokWewers, Mary EllenFrohlich, KatherineMitra, RaktimWeyandt, LisaFrye, Richard E.Mlynarek, StevenWhatley, Art A.Fuchs, ReginaMöhner, MatthiasWherton, JosephFujii, YasuhitoMoise, Imelda K.While, Alison E.Fuller, RichardMollah, Kabirul A.White, AlanFurgal, ChristopherMonge, SusanaWhite, Jason C.Fustinoni, SilviaMons, UteWhite, MathewGagnon, Marie P.Monsees, ThomasWhite, SarahGaines, DavidMontesano, LiusWigg, NeilGalani, IreneMontoya, IvanWilhelm, BarbaraGall, SeanaMontserrat, Javier M.Williams, PrysorGallagher, CarolynMoore, RolandWilliams, DavidGallagher, Carolyn M.Moral, Francisco J.Williams, Rebecca S.Galve, RogerMorales, Aldo W.Williams, SusanGamble, John F.Morency, PatrickWilliamson, TimGao, ChuansiMoreno, Isabel MaríaWillox, Ashlee CunsoloGao, XuboMoriguchi, TakayaWilson, JosephineGarin, BenoitMorin, CoryWilson, LeighGarrett, SueMorleo, MichelaWilson, Mark G.Gary, FayeMorrow-Howell, NancyWindsor, JackGašević, DanijelaMorse, Stephen S.Winquist, AndreaGates, Michael G.Mort, MaggieWithall, JanetGaudart, JeanMorton, Cory M.Wittenberg, EveGautier, AxelMoses, WesleyWoc-Colburn, LailaGbaguidi-Haore, HousseinMovsisyan, NarineWolff, HansGebreab, SamsonMu, LanWolkoff, PederGeller, E. ScottMuggeo, Vito M. R.Wong, BryanGenchi, ClaudioMughini Gras, LapoWong, Hai MingGénéreux, MélissaMuir, SusanWong, Tit-YeeGeorge, David T.Muirhead, ColinWoo, Yin-TakGiatropoulos, AthanassiosMulla, Zuber D.Woodward, AlistairGibson, Chris L.Muller, ClemmaWoolford, Susan J.Gibson, MaggieMuller, Matthew D.Worsley, AnthonyGiddon, Donald B.Mullins, Raywoździak, Anna Z.Gidlöf-Gunnarsson, AnitaMurase, ToshiyukiWright, CaradeeGiesbrecht, NormanMurdoch, David R.Wu, GangGikas, PetrosMuroga, NorihikoWu, JianxinGilbert, PaulMurray, EmilyWu, Kuen-YuhGiles, AudreyMusher-Eizenman, DaraWu, XiangmeiGili, MargaridaNadalin, VictoriaWunder, ChristophGilkey, David P.Nagaraja, T. G.Xu, HaiGill, Jan S.Nagelhout, Gera E.Xu, HowardGillings, MichaelNagler, Rebekah H.Xu, ZhihengGilroy, RoseNakajima, MotohiroYan, GuofenGiné Garriga, RicardNakash, OraYan, PingkunGiraud, EtienneNandakumar, Kutty SelvaYan, TaoGislason, MayaNannette, HoffmanYang, ChunpingGissler, MikaNapoli, ChristianYano, TakahashiGlaesmer, HeideNarita, DaijuYao, WeirongGlockner, KarlNaska, AndronikiYard, Ellen E.Glynn, Peter J.Navarro, RaúlYarmoliuk, TatianaGoarant, CyrilleNeal, April P.Yaworsky, WilliamGoldstein, AdamNearing, Mark A.Yee, DonGomez, Pilar GarciaNemery, BenoitYeh, Gloria Y.Gonçalves, GuilhermeNerín, Cristina.Yeh, Pamela J.Goonetilleke, AshanthaNeupane, SubasYiangou, MinasGoovaerts, PierreNeupane, Sudan PrasadYiannakoulias, NikolaosGordon, KathrynNicolini, RosellaYoons, YoonjinGore, AndreaNiczyporyk, Jowita SamantaYoshizuka, KazuharuGore, DanaNiedzwiedz, Claire L.You, YaqiGorini, GiuseppeNikaj, SildaYuan, Chung-ShinGosselin, PerreNin, JordiYuan, Guo-LiGovaris, AlexanderNiu, BoYumak, ZerrinGrabrucker, AndreasNoonan, DevonZabiegała, BożenaGradoni, LuigiNordell, EvaZajacova, AnnaGrandadam, MarcNovak, NatalijaZander, Kerstin K.Grant, MarcusNull, JanZegers, DoreenGrant, RobertNunes, RaquelZenk, ShannonGrant, William B.Nurmatov, UlugbekZhang, JingjieGranum, Per EinarNyman, AnastasiaZhang, KaiGraudal, Niels AlbertO'Hara, MarilynZhang, Ning JackieGray, SelenaO'Malley, Patrick M.Zhang, ShunGreacen, TimO’Donnell, Deborah A.Zhang, TonglinGreene, NicholasO’Farrell, Patrick J.Zhang, XingyouGreene, ScottOberg, Anna SaraZhang, XuxiangGrigoriev, PavelObregon, Maria-JesusZhang, YulongGrineski, SaraOchoa-Herrera, ValeriaZhang, ZhiyongGrittner, UlrikeOdendaal, WillemZhao, HongtaoGrooten, WimOdland, Jon ØyvindZhao, JinshunGroschup, MartinOehmen, AdrianZheng, W. JimGrossi, EnzoOkechukwu, CassandraZhou, HongjianGrundstein, Andrew J.Olshansky, Stuart J.Zhou, Shang-MingGu, JianweiOnarheim, KristineZhou, YibiaoGuernsey, JudithOng, CorinneZhu, Jin LiangGuirguis, KristenOngerth, Jerry E.Zhu, Xing-QuanGullo, MariaOpdam, PaulZiegler, MatthiasGuo, Jong-LongOpfer, Sarah E.Ziemińska, ElżbietaGupta, AmarOppliger, AnneZierold, KristinaGupta, JhumkaOrdoñana, Juan R.Zima, JanGushulak, Brian D.Ornelas, IndiaZimmerman, Rick S.Gustafsson, SusanneOrtega, ElenaZotter, JeanGuyer, BernardOrtega, RosarioZuckerman, DianaHa, Sang-doOsawa, HarukiZvoleff, Alex


